# Elevation of D-dimer in eosinophilic gastrointestinal diseases in the absence of venous thrombosis: A case series and literature review

**DOI:** 10.1515/med-2024-0960

**Published:** 2024-05-13

**Authors:** Yang Song, Boyu Yang, Wanlei Ren, Doudou Hu

**Affiliations:** Yantai Nurses School of Shandong, Yantai, Shandong, P. R. China; Department of Gastroenterology, Qingdao Hospital, University of Health and Rehabilitation (Qingdao Municipal Hospital), Qingdao, Shandong 266000, P. R. China; Department of Traditional Chinese Medicine, Qingdao Central Hospital, University of Health and Rehabilitation Sciences (Qingdao Central Hospital), Qingdao, Shandong, P. R. China

**Keywords:** eosinophilic gastroenteritis, D-dimer, venous thrombosis, case series, literature review

## Abstract

**Introduction:**

Eosinophilic gastrointestinal diseases (EGIDs) are rare and heterogeneous diseases characterized by excessive eosinophilic infiltration of the digestive system. D-dimer levels and its possible association with disease course were not reported.

**Case series:**

We reported a series of three EGID cases presenting with high levels of D-dimer. No evidence for potential venous thromboembolism was found through computed tomography pulmonary angiogram and vascular ultrasounds. Moreover, D-dimer levels decreased after short-time systemic prednisolone administration, accompanied by remission of clinical symptoms and decrease of peripheral eosinophil counts and IgE levels.

**Conclusion:**

Elevation of D-dimer in EGID may not represent thrombotic events but is possibly associated with disease severity. More population-based studies are needed to delineate the potential relationship among D-dimer, thrombosis, and inflammation in EGID.

## 1 Introduction

Eosinophilic gastrointestinal diseases (EGIDs) are characterized by excessive infiltration of eosinophils in the gastrointestinal tract, ranging from the esophagus to the rectum, which includes eosinophilic esophagitis (EoE), eosinophilic gastritis (EoG), eosinophilic enteritis, eosinophilic colitis (EoC), etc. [1]. Patients suffer from different types of symptoms depending on the involved segments, including nausea, abdominal pain, diarrhea, etc. [2]. Most patients are obliged to corticosteroid therapy accompanied by various side effects, such as metabolic disorders [3]. Moreover, more than half of the patients suffer persistent disease activity or a relapsing and remitting course.

Laboratory results in EGID are mostly unspecific, including eosinophilia [4], iron-deficiency anemia [5], elevation of serum IgE [6], etc. However, abnormalities of coagulation parameters are not reported in previous cases. In the coagulation process, D-dimer is derived from fibrin degradation products (FDPs) when plasmin cleaves insoluble fibrin monomers [7]. It has been widely used in the identification, evaluation, and treatment effects of venous thromboembolism (VTE) and various artery diseases [8]. However, role of D-dimer in EGID and its possible association with VTE are not reported. Thus, we report a case series of EGID with an uncommon presentation of D-dimer elevation in the absence of VTE.

## 2 Case series

### 2.1 Case 1

A 26-year-old Chinese man, with no significant past medical history, presented to the clinic with 3-day history of abdominal pain localized to the epigastrium. He denied any recent travel, sick contact, or drug administration prior to the onset of symptoms. The physical examination findings were unremarkable.

The total white blood cell count was 10,840/µL (3,500–9,500/µL, reference range in our hospital), and eosinophil count was 5,130/µL (20–520/µL). The fecal occult blood test was weakly positive. The level of immunoglobin (Ig) E was 220 IU/mL (≤100 IU/mL) and the patient was weakly allergic to peanut and soya bean. D-dimer level was three times more than the normal range: 1.57 µg/mL (0–0.5 µg/mL). Liver and renal function, serum tumor markers, lipid levels, thyroid function, as well as immunity and inflammation-related markers, such as IgG, IgG4 isotype, IgA, IgM, erythrocyte sedimentation rate, antinuclear antibodies, and C-reactive protein were within the normal range.

**Figure 1:**
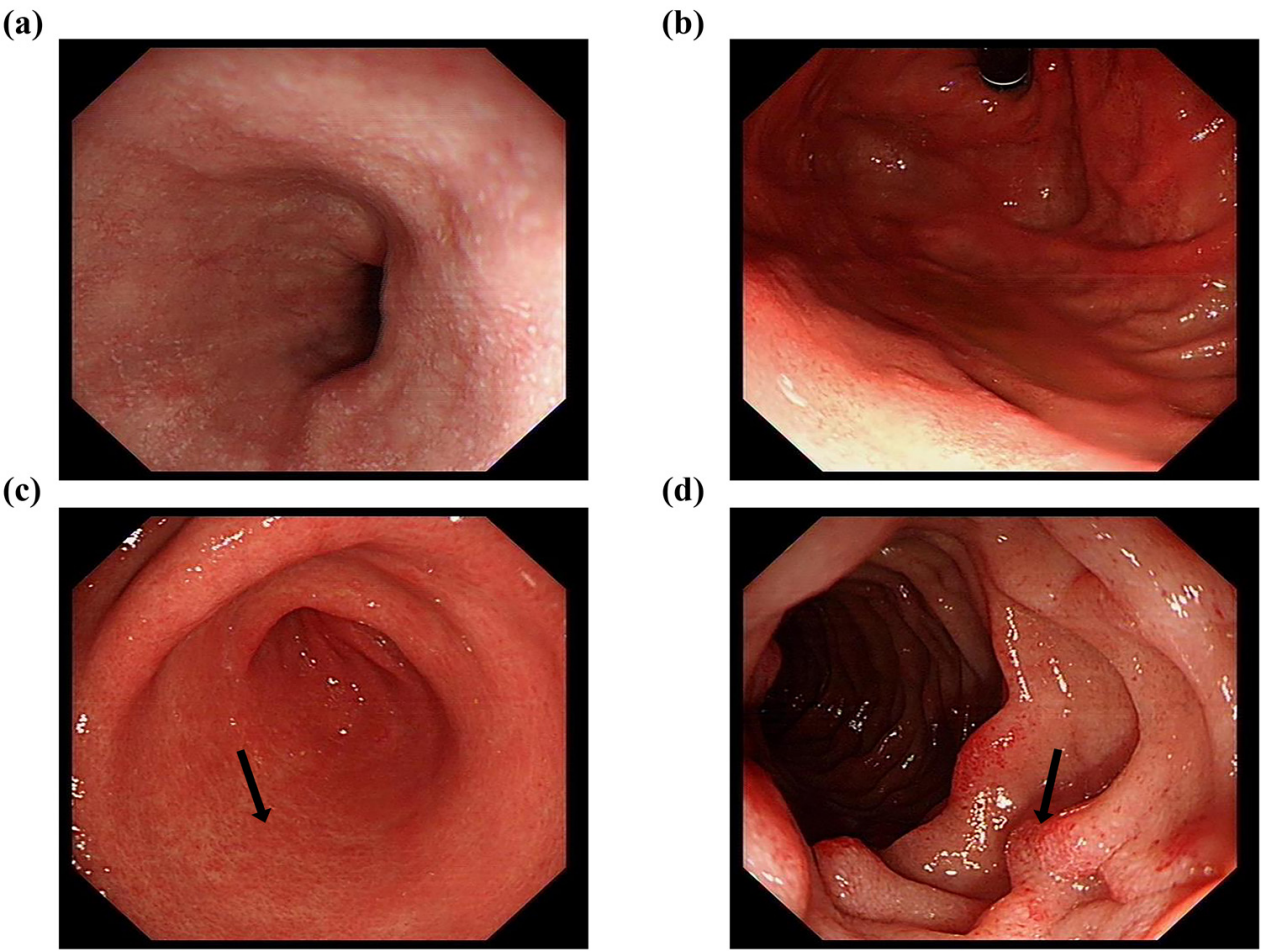
Representative images of upper endoscopy indicating scattered flat hyperemia and erosion of the gastric antrum and duodenum. (a) Esophagus, (b) gastric fundus, (c) gastric antrum, and (d) duodenum.

**Figure 2:**
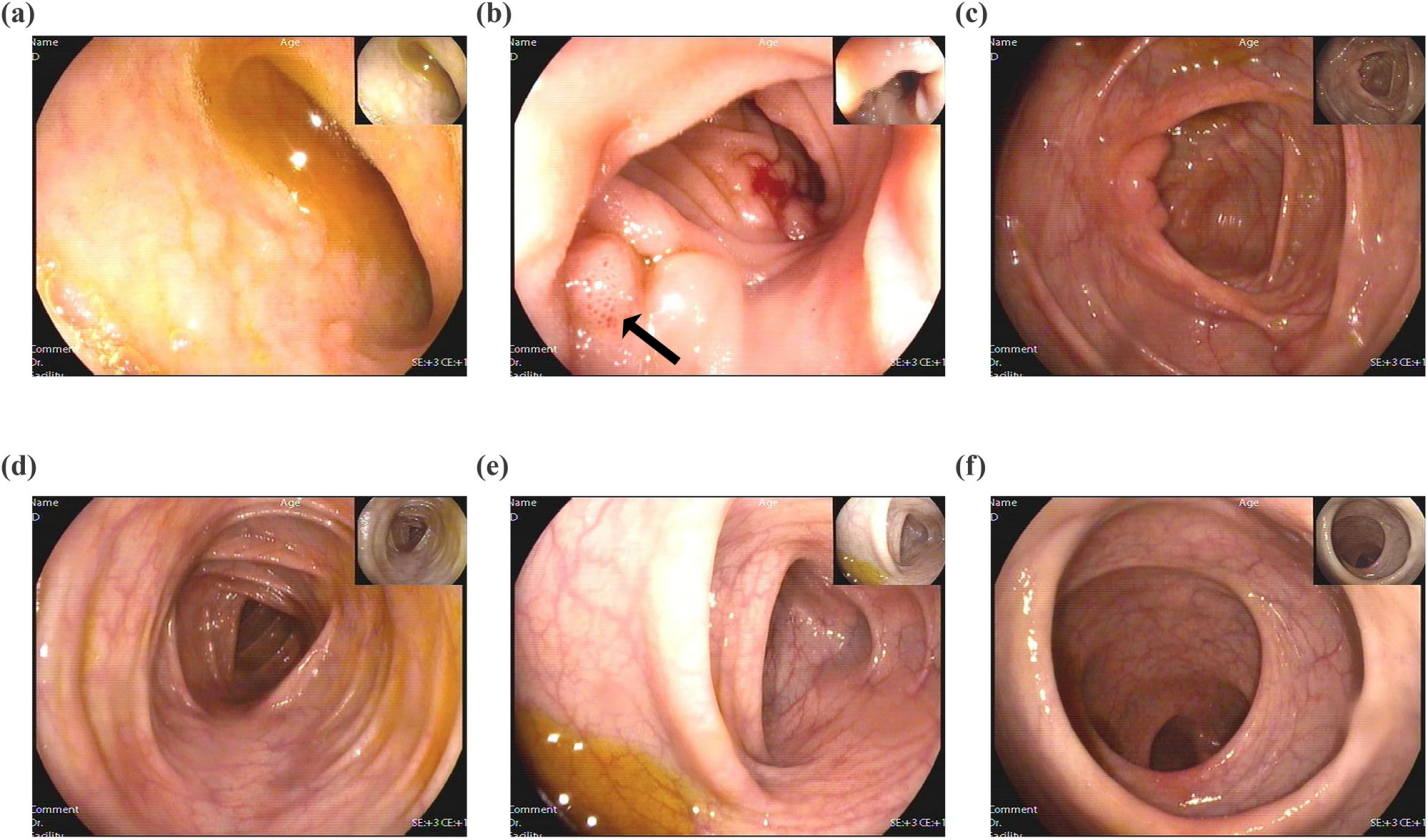
Representative images of colonoscopy indicating scattered punctate erosion of the terminal ileum. (a) Terminal ileum, (b) terminal ileum, (c) ileocecal region, (d) transverse colon, (e) descending colon, and (f) rectum.

**Figure 3:**
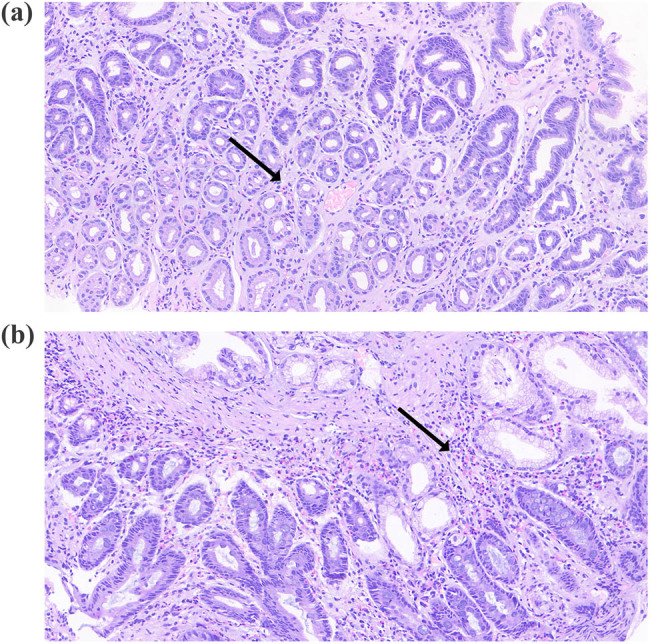
Histological images of the gastric antrum showing infiltration of eosinophils. (a) and (b) gastric antrum.

A contrast-enhanced computed tomography (CT) scan of the chest and abdomen showed diffuse circumferential thickening of the esophagus and gastric antrum. The lung, liver, gall bladder, bile duct, spleen, and pancreas showed no abnormalities and there was no lymph node enlargement. Upper endoscopy revealed scattered flat hyperemia and erosion of the gastric antrum and duodenum (Figure 1). Colonoscopy showed scattered punctate erosion of the terminal ileum (Figure 2). Histological analysis showed moderately active gastritis and duodenitis, with more than 30 eosinophils per high-power field (HPF) in the gastric antrum (Figure 3). Differential diagnosis was also made according to current examinations. The patient reported no travel history. Stool tests for hookworm, whipworm, ascaris, and amoeba were negative. No evidence of parasitic infection was found. 13C-urea breath test and pathology analysis for *Helicobacter pylori* were negative. The patient also denied a history of medications, including mycophenolate and non-steroidal anti-inflammatory drugs. The diagnosis of EoG was validated.

**Figure 4:**
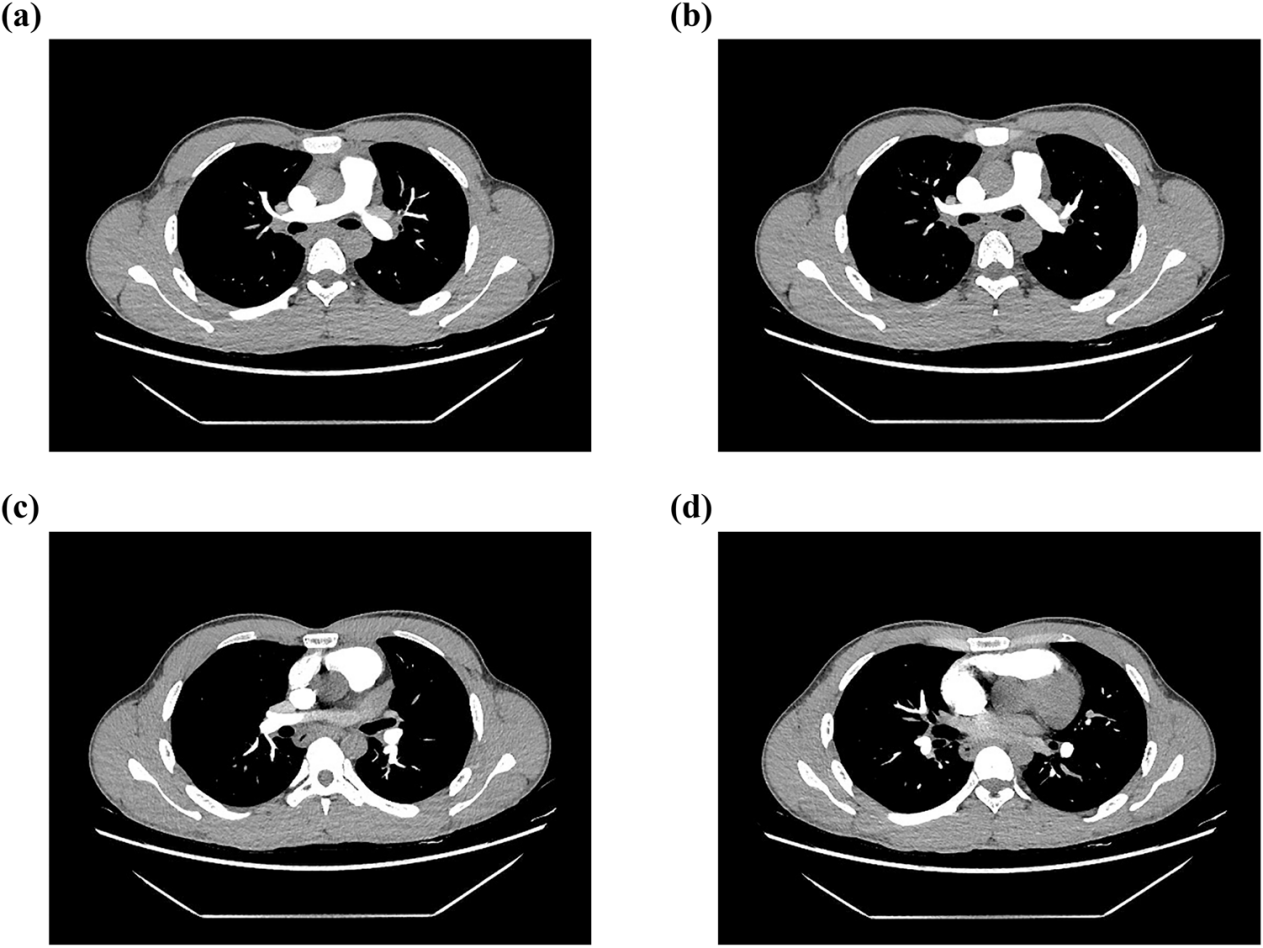
Representative images of CTPA showing no thrombosis. (a) to (d), different slices of CTPA.

We wondered whether D-dimer elevation was a hint for venous thrombosis. However, computed tomography pulmonary angiogram (CTPA, Figure 4), vascular ultrasound for lower extremity, and mesenteric vessels were negative for possible thrombosis. Upon diagnosis, the patient was administered systemic steroid therapy (prednisolone 40 mg/day) followed by rapid symptomatic improvement. Five days after steroid administration, the eosinophil count was rapidly decreased to 590/µL and the D-dimer level was also within the normal range: 0.22 µg/mL. IgE was also downregulated to 180 IU/mL. The patient was discharged for further monitoring and follow-up.

### 2.2 Case 2

A 67-year-old female was referred to our department for abdominal pain over 10 days. She also had a distended abdomen and diarrhea. Her past medical history was significant for hypertension and her only surgical history was appendectomy due to appendicitis. She was taking valsartan to treat hypertension. Vital signs were stable and physical examination findings were not significant.

Labs were notable for a white cell count of 24,870/µL and an eosinophil count of 17,970/µL. The fecal occult blood test was also positive. The level of IgE was more than the upper limit 1,130 IU/mL and she was also weakly allergic to soybeans. Her lipid levels were abnormal with total triglyceride being 2.37 mmol/L and total cholesterol being 5.81 mmol/L. The D-dimer level was also more than the normal range: 0.8 µg/mL. Liver and renal function, serum tumor markers, and immunity-related markers were within normal range.

CTPA, vascular ultrasound for the lower extremity, and mesenteric vessels also showed no potential thrombosis. CT scan of the chest and abdomen showed cholecystitis and a small amount of pelvic effusion. Upper endoscopic findings indicated schistose erythema and erosion of the gastric antrum and duodenum. Histology of gastric antrum suggested massive infiltration of eosinophils (approximately 35 cells/HPF). Colonoscopy showed no abnormal findings. The patient also had no travel history. Stool tests were negative for parasitic infection. 13C-urea breath test and pathology analysis for *H. pylori* were negative. Apart from valsartan, the patient denied the current administration of medications. Diagnosis of EoG was verified.

She was administered with prednisolone 20 mg/day. Following the corticosteroid prescription, abdominal pain was soon relieved. Seven days after the prednisolone prescription, peripheral eosinophils were 2,460/µL. IgE level was decreased to 835 IU/mL. D-dimer concentration was 0.23 µg/mL.

### 2.3 Case 3

A 28-year-old Congolese male complained of symptoms of epigastric pain, abdominal distension, and acid reflux for 20 days, which were deteriorated after meals. No significant past medical history was reported and physical examination was unremarkable.

The peripheral blood profile showed a leukocytosis (11,540/µL) with 42.5% eosinophils (4,900/µL). IgE level was slightly high (156 IU/mL). Serum CA-125 was over normal range: 122.71U/ml. A high concentration of D-dimer (19.59 µg/mL) was also observed. Venous thrombosis was preliminarily excluded through CTPA and vascular ultrasound for mesenteric vessels and lower extremity. Chest and abdominal CT scans revealed diffuse thickening of the small intestine, ascites, and pleural effusion. No solid mass was observed. Upper endoscopy showed punctate erythema and erosion of the esophagus and gastric corpus, diffuse mucosal edema, and erosion of gastric angle and antrum. Histological analysis showed severely active gastritis and duodenitis with eosinophil infiltration more than 50/HPF in the gastric antrum and descending part of duodenum. He refused to undergo a colonoscopy.

The patient was prescribed prednisolone 30 mg/day to induce clinical remission. Shortly after corticosteroid administration, he reported significant alleviation of symptoms of abdominal pain, distension, and acid reflux. Five days after treatment, the peripheral eosinophil count was reduced to the normal range: 50/µL. D-dimer level was markedly decreased to 0.63 µg/mL. IgE level was within normal range. The peripheral eosinophil count, D-dimer, and IgE levels of all three cases before and after corticosteroid treatment are displayed in Table 1.

**Table 1:** Peripheral eosinophil count, D-dimer, and IgE levels of all three cases before and after corticosteroid treatment

	At admission			After treatment (5–7 days)		
	Peripheral eosinophil count (cell/µL)	D-dimer level (µg/mL)	IgE level (IU/mL)	Peripheral eosinophil count (cell/µL)	D-dimer level (µg/mL)	IgE level (IU/mL)
Case 1	5,130	1.57	220	590	0.22	180
Case 2	17,970	5.81	1,130	2,460	0.23	835
Case 3	4,900	19.59	156	50	0.63	74


**Informed consent:** Informed consent has been obtained from all individuals included in this study.

## 3 Discussion

The overall prevalence of EoG and EoE is 5.1/100,000 persons, and the prevalence rate of EoC is 2.1/100,000 in the US [9], which makes EGID still a rare disease. No consensus has been reached concerning diagnostic criteria for EGID and patients showed various clinical symptoms and disease courses. In some cases, uncommon clinical presentations could be observed. Here, we reported a case series of EGID with elevation of D-dimer in the absence of VTE. D-dimer levels decreased after short-time prednisolone administration, with the remission of clinical symptoms and a decrease of peripheral eosinophil counts and IgE levels.

During coagulation progress, plasmin cleaves cross-linked insoluble fibrin monomers to yield by-products known as FDPs [10]. Among these products, the remnant composed of two adjacent bonded D-domains from cross-linked fibrils are what are now known as “D-dimers” [11]. Serum D-dimer levels correlate with various pathophysiological conditions. One of the critical roles is the diagnosis of VTE [12]. Increased D-dimer levels indicated highly possible active VTE, especially when D-dimer assays were performed shortly after symptom onset [13]. The sensitivity, specificity, and positive predictive value of D-dimer concentration greater than 500 ng/mL for acute pulmonary emboli were 93.3, 25.0, and 30.4%, respectively [14]. To rule out possible thrombosis, all three cases underwent CTPA, as well as vascular ultrasound for mesenteric vessels and lower extremity. All results were negative, which indicated that thrombosis may not be the appropriate explanation for D-dimer elevation.

D-dimer was also associated with the onset and disease course of various cardiovascular diseases. In acute aortic dissection (AAD), D-dimer testing provided high sensitivity and a negative likelihood ratio. At a cut-off level of 0.9 and 0.1 µg/mL, negative predictive value (NPV) achieved 92% and 100%, respectively [15]. Similar results confirming the high NPV of D-dimer levels in AAD were also demonstrated by Cui et al. [16]. Plasma D-dimer was also strongly association with aortic aneurysm (AA) for cut-offs 400 and 900 ng/mL, respectively, and its high concentration (>900 ng/mL) correlated with a faster aneurysmal growth rate [17]. However, the role of D-dimers in the diagnosis and risk stratification of coronary artery disease (CAD) patients remains disputable. The potential hypothesis may be that the underlying coronary atherosclerotic activity may lead to fibrinolysis, and a subsequent increase in plasma D-dimer levels [18]. Its increase might precede the onset of acute coronary syndrome (ACS). In this case series, all three cases were excluded for AAD and AA based on symptoms, chest CT scan, and CTPA. Electrocardiogram and classic cardiac biomarkers such as troponin and creatine kinase MB also did not support ACS diagnosis. Moreover, the rapid decrease in D-dimer concentration after corticosteroid administration was not consistent with CAD or ACS disease course.

Inflammation has been recognized as a regulator of coagulation and fibrinolytic system [19,20]. Acute inflammation is known to shift the hemostatic balance toward a prothrombotic and antifibrinolytic state in which there is an increase in circulating levels of several key procoagulant and antifibrinolytic mediators. In HIV patients, D-dimer levels were markedly decreased in antiretroviral therapy (ART) compared to untreated patients [21]. Stopping ART in primary HIV patients leads to IL-6 and D-dimer significantly increase in pre-treatment concentrations [21]. Besides, in type 1 diabetic patients, serum D-dimer levels showed a significant positive correlation with TNF-α concentrations, indicating a possible interrelationship between inflammation and hypercoagulability status [22]. The potential explanation might be that proinflammatory cytokines, such as IL-6 and TNF-α, can stimulate the release of pro-coagulant molecules and inhibit the expression of anti-coagulant molecules by endothelial cells, resulting in a hypercoagulability state [23]. However, such studies were not reported in EGID and the correlation needed further validation in large-scale population studies.

In conclusion, we reported a case series of EGID with elevation of D-dimer in the absence of VTE. D-dimer levels decreased after short-time prednisolone administration, with a remission of clinical symptoms and a decrease in peripheral eosinophil counts and IgE levels. Further large-scale population-based studies are warranted to validate D-dimer levels in EGID.
